# Investigation of the Expression of Myogenic Transcription Factors, microRNAs and Muscle-Specific E3 Ubiquitin Ligases in the Medial Gastrocnemius and Soleus Muscles following Peripheral Nerve Injury

**DOI:** 10.1371/journal.pone.0142699

**Published:** 2015-12-21

**Authors:** Rebecca Wiberg, Samuel Jonsson, Liudmila N. Novikova, Paul J. Kingham

**Affiliations:** 1 Department of Integrative Medical Biology, Section of Anatomy, Umeå University, Umeå, Sweden; 2 Department of Surgical & Perioperative Sciences, Section of Hand and Plastic Surgery, Umeå University, Umeå, Sweden; University of Minnesota Medical School, UNITED STATES

## Abstract

Despite surgical innovation, the sensory and motor outcome after a peripheral nerve injury remains incomplete. One contributing factor to the poor outcome is prolonged denervation of the target organ, leading to apoptosis of both mature myofibres and satellite cells with subsequent replacement of the muscle tissue with fibrotic scar and adipose tissue. In this study, we investigated the expression of myogenic transcription factors, muscle specific microRNAs and muscle-specific E3 ubiquitin ligases at several time points following denervation in two different muscles, the gastrocnemius (containing predominantly fast type fibres) and soleus (slow type) muscles, since these molecules may influence the degree of atrophy following denervation. Both muscles exhibited significant atrophy (compared with the contra-lateral sides) at 7 days following either a nerve transection or crush injury. In the crush model, the soleus muscle showed significantly increased muscle weights at days 14 and 28 which was not the case for the gastrocnemius muscle which continued to atrophy. There was a significantly more pronounced up-regulation of MyoD expression in the denervated soleus muscle compared with the gastrocnemius muscle. Conversely, myogenin was more markedly elevated in the gastrocnemius versus soleus muscles. The muscles also showed significantly contrasting transcriptional regulation of the microRNAs miR-1 and miR-206. MuRF1 and Atrogin-1 showed the highest levels of expression in the denervated gastrocnemius muscle. This study provides further insights regarding the intracellular regulatory molecules that generate and maintain distinct patterns of gene expression in different fibre types following peripheral nerve injury.

## Introduction

Both prolonged axotomy and prolonged denervation can influence the extent of functional recovery which can be achieved after a peripheral nerve injury [1, 2]; in the latter case deterioration of the intramuscular nerve sheaths results in failure to attract and provide support for the regenerating axons [2]. Furthermore, following reinnervation, long term denervated muscle fibres fail to recover entirely from atrophy, most likely as a result of reduced satellite cell (SCs) numbers and impaired SCs activity levels [3]. Moreover, muscle regeneration is severely impaired by denervation-induced deposits of extracellular matrix and the spatial separation of SCs [4]. Fu et al [1] described that prolonged denervation is very detrimental regarding the functional recovery after a peripheral nerve injury and accounts for a 90% reduction in the number of functional motor units, compared with a 30% reduction after prolonged axotomy at the same time point, which highlights the importance of the target organ as a critical factor regarding the final outcome of a peripheral nerve injury.

Based on the expression of the myosin heavy chain (MyHC) gene, it is possible to define four different types of muscle fibres including type I, IIa, IIx and IIb [5], which diverge along a continuum of contraction speed and endurance. Type I is slow contracting, with a high capacity for oxidative metabolism and good endurance and type IIb fibres are fast contracting, fatigable and mainly dependent on glycolytic metabolism. Thus, fast and slow fibres contain fast and slow MyHC isoforms that display high or low actin-dependent ATPase activity, respectively [6]. Depending on the biochemical and physiological properties of the muscle it is more, or less, vulnerable to various types of insult, and studies suggest that the muscle phenotype may influence the disease progression [7]. Previously, we showed in a sciatic nerve injury model with delayed repair, that the size of fast type fibres was significantly reduced after one month delayed repair, whilst the slow type fibres were not significantly reduced in size until 6 month delayed repair [8].

Advances in molecular biology have highlighted the potential role of microRNAs (miRNAs) in influencing clinical outcomes following peripheral nerve injuries [9]. miRNAs are a class of small, ∼22 nucleotides long non-coding single stranded RNAs, that negatively regulate gene expression through post-transcriptional inhibition by complementary base-pair binding of the miRNA seed sequence (2–7 nucleotides) in the 3´untranslated region of target mRNAs [9, 10]. miRNAs down regulate gene expression by two different mechanisms, translational repression and mRNA degradation [9, 10], which is dependent on the degree of complementarity. Thus, when a microRNA imperfectly pairs to its target mRNA, translational repression is thought to be the primary mechanism of action, while mRNA cleavage is thought to take place when miRNA perfectly pairs to the targeted mRNA [9, 10]. Since the requirement for target complementarity is only partial, one single miRNA can potentially control hundreds of target genes and each mRNA can be regulated by several different miRNAs [11, 12].

Both central and peripheral axons contain miRNA and it has been demonstrated that the miRNA biosynthetic machinery responds to peripheral nerve lesions in an injury regulated pattern; at least 20–30% of human protein coding genes are modulated by miRNAs [12]. Thus, miRNA exerts a complex network of negative gene regulation but the physiological significance of these pathways remains to be elucidated. The muscle specific miRNAs, miR-1 and miR-206, together with several non-muscle specific microRNAs, are required for muscle proliferation and differentiation through interaction with myogenic factors. MyoD, myogenin, MRF4 and myf5, all myogenic regulatory factors (MRFs), form a family of muscle-specific basic helix-loop-helix (bHLH) transcription factors that govern differentiation of muscle cells during development [13, 14]. MyoD and myogenin regulate differential muscle gene expression [15] and in the adult muscle, MyoD is highly expressed in fast muscle fibres [16, 17] and regulates fast muscle development [17]. Conversely, myogenin is highly expressed in slow muscle fibres [16]. Each of the MRFs is capable of activating muscle-specific gene expression, yet distinct functions have not been ascribed to the individual proteins. Myogenin and MyoD are transcriptional regulators of miR-206 [18, 19]. Thus there is increasing evidence that the interaction between microRNAs and myogenic transcription factors may influence the outcome of nerve injuries. Furthermore, Forkhead box O transcription factors (Foxo) [20] and muscle-specific E3 ubiquitin ligases, such as muscle RING-finger 1 (MuRF1) and Atrogin-1/muscle atrophy F-box (MAFbx) [21], which mediates protein degradation through the ubiquitin proteasome system (UPS), are critical players regarding the functional outcome of the target organ following peripheral nerve injury.

By studying the expression of myogenic transcription factors, muscle specific microRNAs and muscle-specific E3 ubiquitin ligases at several time points following denervation in two different muscles, the gastrocnemius and soleus muscles, containing mainly fast and slow muscle fibres respectively, we aimed to gain more insight about how these molecular pathways influence the degree of atrophy following denervation.

## Materials and Methods

### Experimental animals and ethics statement

This study was approved by the Northern Swedish Regional Committee for Ethics in Animal Experiments at Umeå University (protocol number A186-12). Adult (10–12 weeks old) inbred female Sprague Dawley rats and adult (8–10 weeks old) inbred female Fisher F344 rats (Scanbur BK AB, Sweden) were used in this study. The animal husbandry was in accordance to the standards and regulations provided by the National Institutes of Health Guide for Care and Use of Laboratory Animals (NIH Publications No. 86–23, revised 1985) and the European Communities Council Directive (86/609/EEC). Surgery was performed aseptically under general anesthesia using a mixture of Ketamine (Ketalar 50 mg/ml Pfizer, Sweden) and Xylazine (Rompun 20 mg/ml Bayer Health Care, Germany) by intraperitoneal injection. Finadyne (Schering-Plough Animal health 50 mg/ml) was administered post-operatively. Rats and their well-being were observed throughout the experimental period.

### Surgical procedures and experimental groups

The animals were divided into the following experimental groups: (i) sciatic nerve crush injury (total n = 20, 5 animals for each time point), (ii) sciatic nerve transection without repair (total n = 20, 5 animals for each time point), (iii) sciatic nerve transection with immediate nerve repair (n = 5) and (iv) sciatic nerve transection with delayed nerve repair (n = 14, 5 animals in the 1 and 3 month delayed repair group and 4 animals in the 6 month delayed repair group). The group that served as a control were un-operated animals (4 animals).

The sciatic nerve was exposed by bluntly dividing the gluteal muscles of the thigh. Under an operating microscope (Zeiss, Carl Zeiss, Germany) the nerve was either crushed with a fine aortic clamp for 10 s or transected at a standardized distance from the spinal cord, approximately 5 mm from the most proximal branch of the sciatic nerve. In the group with sciatic nerve transection without repair the nerves were then capped to prevent distal reinnervation. Caps were made out of polyethylene tubes and each nerve stump was introduced and anchored to the cap using the 10–0 Ethilon suture. In the group with immediate nerve repair the nerve stumps were bridged with a 10 mm sciatic reversed autograft. In the group with delayed nerve repair after transection the nerve stumps were ligated approximately 1 mm from the cut end using an 8–0 nonresorbable Ethilon suture and capped as above to prevent reinnervation. The proximal stump was put under the femoral quadriceps muscle and the distal stump introduced into the popliteal fossa. After 1, 3 or 6 months, the wound was re-opened and the proximal and distal stumps of the sciatic nerve were re-exposed, trimmed by 2–3 mm to remove neuroma/scar tissue and repaired using a 10 mm sciatic reversed sciatic nerve graft from a donor Fisher rat. The graft was fixed with four interrupted epineurial sutures aligned circumferentially in each anastomosis using micro instruments and a 10–0 nonresorbable Ethilon suture. After surgery the wound was closed in layers.

The operated animals were allowed to survive for 1, 7, 14 and 28 days in the groups with sciatic nerve crush injury and sciatic nerve transection without repair and 13 weeks after immediate or delayed nerve repair.

### Tissue processing

At the end of the survival period the rats were terminally injected with an intraperitoneal overdose of sodium pentobarbital (240 mg/kg, Apoteksbolaget, Sweden). The medial gastrocnemius muscles and the soleus muscles from operated ipsilateral side and non-operated contralateral side were harvested, weighed and fast frozen in liquid nitrogen.

### Quantitative RT-PCR (qRT-PCR)

Total RNA was isolated from muscles using a miRNeasy mini kit (Qiagen, Sweden) and the purified RNA was quantified by determining the absorbance at 260 nm using a Nanodrop 2000/2000c spectrophotometer (ThermoScientific, Sweden). Muscle samples from rats in each experimental group were pooled and 1–5 ng total RNA was converted to cDNA using a First-Strand cDNA Synthesis Kit (Bio-Rad iScript cDNA synthesis kit). The reaction mix was incubated in a thermal cycler under the following conditions; 5 min at 25°C, 30 min at 42°C and finally 5 min at 85°C. qRT-PCR was subsequently performed using SsoFastTM EvaGreen Supermix (BioRad) according to the manufacturer recommendations (enzyme activation at 95°C for 30 s followed by up to 40 cycles of denaturation (95°C for 5 s) and annealing/extension (5s at optimal temperatures shown below). A GeNorm bioinformatic analysis (integrated in qbasePLUS software, Biogazelle NV, Belgium) of multiple commonly used housekeeping genes showed that actin, SDHA and HSPCB were the best combination to use for normalisation when calculating gene expression changes for both muscle types and injury models upto 28 days. 18S was used in all other experiments. MyoD forward 5ʹ-TGTAACAACCATACCCCACTC-3ʹ and reverse 5ʹ-AGATTTTGTTGCACTACACAG-3ʹ primers with annealing temperature of 60.6°C, myogenin forward 5ʹ-CACATCTGTTCGACTCTCTTC-3ʹ and reverse 5ʹ-ACCTTGGTCAGATGACAGCTT-3ʹ primers with annealing temperature of 58°C, Atrogin forward 5´-GAACAGCAAAACCAAAACTCAGTA-3´ and reverse 5´-GCTCCTTAGTACTCCCTTTGTGAA-3´ primers with annealing temperature of 60°C, MuRF forward 5´-TGTCTGGAGGTCGTTTCCG-3´ and reverse 5´-ATGCCGGTCCATGATCACTT-3´ primers with annealing temperature of 64°C, Actin forward 5′-ACTATCGGCAATGAGCGGTTC-3′ and reverse 5′-AGAGCCACCAATCCACACAGA-3′ primers with annealing temperature of 65°C, SDHA forward 5′-AGACGTTTGACAGGGGAATG-3′ and reverse 5′-TCATCAATCCGCACCTTGTA-3′ primers with annealing temperature of 60.9°C, HSPCB forward 5′-GATTGACATCATCCCCAACC-3′ and reverse 5′-CTGCTCATCATCGTTGTGCT-3′ primers with annealing temperature of 61.9°C and 18S forward 5′-TCAACTTTCGATGGTAGTCGC-3′ and reverse 5′-CCTCCAATGGATCCTCGTTAA-3′ primers with annealing temperature of 61.4°C were purchased from Sigma, UK.

### MicroRNA

Total RNA was prepared as above and muscle samples from rats in each experimental group were pooled and 1–10 ng total RNA was converted to cDNA using a TaqMan^®^ MicroRNA Reverse Transcription Kit (Taqman^®^ Small RNA Assays). The reaction mix was incubated in a thermal cycler under the following conditions; 30 min at 16°C, 30 min at 42°C and finally 5 min at 85°C. qRT-PCR was subsequently performed using Taqman^®^ Universal PCR Master Mix according to the manufacturer recommendations (enzyme activation at 95°C for 10 min followed by up to 40 cycles of denaturation (95°C for 15 s) and annealing/extension (60°C for 60 s). The Taqman^®^ MicroRNA assays, hsa-miR-206 and rno-miR-1, were purchased from Invitrogen, Sweden.

### Muscle morphological analysis

7–12μm thick transverse sections of gastrocnemius and soleus muscles from the contra-lateral and operated sides were cut on a cryostat. Sample were either stained with haematoxylin and eosin or fixed with 4% (w/v) paraformaldehyde for 15 min and then blocked with normal serum prior to immunostaining. 12μm thick sections were incubated with monoclonal primary antibodies raised against either fast and slow myosin heavy chain protein (NCL-MHCf and NCL-MHCs, Novocastra, UK both 1:20 dilution) for 2 h at room temperature together with rabbit anti-laminin antibody (Sigma; 1:200 dilution). 7μm thick sections were stained with rabbit anti-dystrophin antibody (GeneTex; 1:5000 dilution) and either mouse anti-MyoD antibody (BD Pharmingen; 1:200 dilution) or mouse anti-myogenin antibody (abcam; 1:200 dilution). After rinsing in phosphate-buffered solution, secondary goat anti-rabbit and goat anti-mouse antibodies either Alexa Fluor 488 or Alexa Fluor 568 conjugated (Invitrogen; 1:200 dilution) were applied for 1 h at room temperature in the dark. The slides were cover-slipped with Prolong anti-fade mounting medium containing DAPI. The staining specificity was confirmed by omission of primary antibodies. Preparations were viewed under an Eclipse80i fluorescence microscope and images captured with Nikon Elements Imaging Software.

Morphometric analysis of muscle sections was performed on coded slides without knowledge of their source. Five random fields were chosen (using the 20X objective) and images for the immunolocalisation of each myosin heavy chain type plus that for laminin were captured using the appropriate emission filters, and combined to provide dual-labelled images. Each image contained at least 10 individual muscle fibres for analysis. Image-Pro Plus software was calibrated to calculate the mean area in μm for each muscle. The injured side was expressed relative to the contra-lateral control side and the relative mean % ± SEM calculated for each group.

### Statistical analysis

In order to determine the statistical difference between groups, one-way analysis of variance (ANOVA) complemented by Bonferroni test (Prism Graph-Pad software) was used. Two-way ANOVA was also used to further compare soleus and gastrocnemius muscles in each of the analyses. Statistical significance was set as *p<0.05, **p<0.01, ***p<0.001.

## Results

### Morphological changes and atrophy of muscle after nerve injury

**Fig 1:**
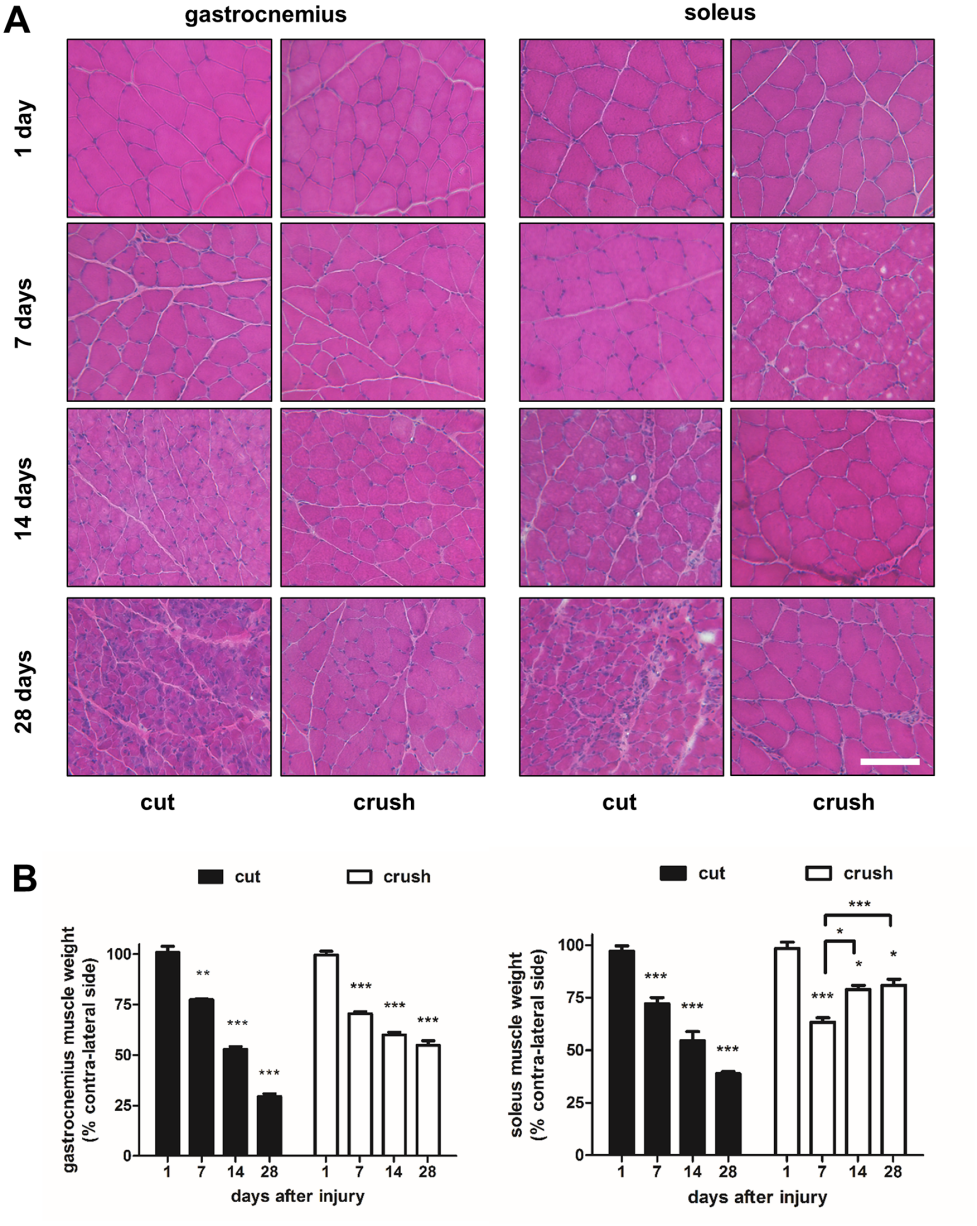
Muscle morphology and quantification of weights. Gastrocnemius and soleus muscles were harvested at 1, 7, 14, 28 days following either sciatic nerve transection (cut) injury or crush injury. **(A)** Sections of muscle tissue were stained with haematoxylin and eosin. **(B)** At the time of harvest, the contra-lateral and operated muscle gastrocnemius and muscle soleus were weighed. Data are expressed as percentage of contra-lateral side weights after nerve transection (cut) and crush injury 1, 7, 14 and 28 days after nerve insult. Statistically significant differences compared with the contra-lateral side are represented by *P< 0.05, **P< 0.01, ***P< 0.001. Connecting bars also show *P<0.05, ***P<0.001 for soleus crush injury at 7 days v’s 14 days and 28 days respectively. Two-Way ANOVA indicates gastrocnemius and soleus are significantly different (P<0.001) across the time course post-crush injury.

The medial gastrocnemius and soleus muscles harvested at various time points following either nerve transection or nerve crush injury were stained with haematoxylin and eosin (Fig 1). As early as 7 days after injury there were noticeable morphological changes, the muscle fibres showed a more rounded shape compared with the characteristic mosaic pattern still evident at day 1. The muscles harvested 28 days after nerve transection injury showed the greatest signs of morphological atrophy; small muscle fibres were surrounded by large numbers of other cells, presumably inflammatory cells and fibroblasts. In the animals treated with nerve crush injury, the morphological changes were less marked at 28 days. Interestingly, the soleus muscles 28 days after crush injury looked almost identical to the muscles 1 day after injury, suggesting a significant recovery with time (Fig 1).

Animals undergoing sciatic nerve transection showed a progressive decline in the medial gastrocnemius wet weight over the period of 1–28 days post-injury (Fig 1). By day 28, there was a 70.57% ± 1,39 reduction in the weight of the operated side compared with the contra-lateral side (Fig 1). Animals undergoing crush injury, showed much less loss of muscle weight (45.19% ± 2.6 at day 28). There was a similar progressive weight loss in the soleus muscle of animals with sciatic nerve transection (Fig 1). In contrast, in the crush injury treated animals, after an initial reduction in weight by 36.43% ± 2.67, the weight of the soleus muscles recovered and showed just 19.13% ± 2.90 loss at day 28 (Fig 1). These results suggested that the soleus, a predominantly slow fibre type muscle, was less susceptible to injury (showing a recovery from day 7), so we investigated whether there were any specific differences between fast type and slow type fibres in the mixed fibre type gastrocnemius muscle.

**Fig 2:**
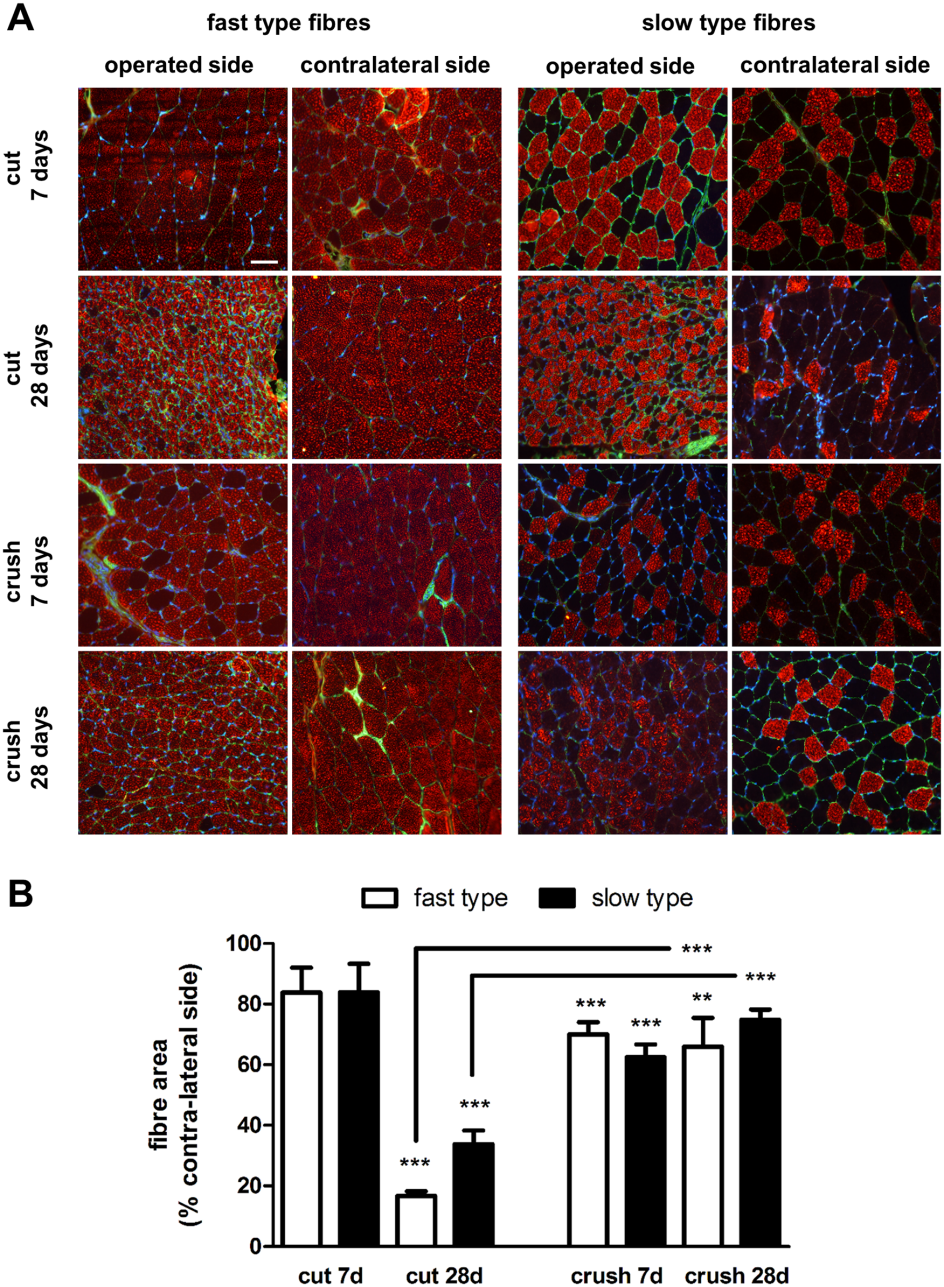
Fast and slow type gastrocnemius muscle fibre morphology. **(A)** Transverse sections of contra-lateral and operated side muscles were stained with laminin antibody (green) and either fast type or slow type myosin heavy chain protein antibody (red). Samples shown are from animals undergoing sciatic nerve transection (cut) or crush injury 7 and 28 days after nerve injury. Scale bar = 50 μm. **(B)** Quantification of muscle fibre size. Computerised image analysis was used to calculate the mean ± SEM area (μm^2^) of fast type and slow type fibres in muscle obtained from the contra-lateral and operated sides of animals undergoing sciatic nerve transection (cut) and crush injury 7 and 28 days after insult. Data are expressed as percentage of the contra-lateral side. *P< 0.05, **P< 0.01, ***P< 0.001. Connecting bars also show ***P<0.001 for fast type and slow type fibres 28 days after cut and crush injury respectively.

Contra-lateral muscles from animals undergoing nerve transection or crush injury followed by 7 or 28 days without repair showed a well-organized structure, predominantly populated by fast type muscle fibres (Fig 2). The muscles from the operated side of animals undergoing nerve transection showed a much more affected morphology and an apparent reduction in muscle fibre size compared to the operated side muscles from animals undergoing crush injury, especially in respect to fast type fibres (Fig 2). Furthermore, following nerve transection indication of muscle fibre grouping was observed with time. Quantitative analysis showed that the animals undergoing sciatic nerve transection exhibited a 16.20% ± 8.26 and 83.25% ± 1.48 reduced fast type fibre area in the gastrocnemius muscle 7 and 28 days after injury respectively (Fig 2). There was also a reduction in size in the slow type fibres, but to a lower extent (66.25% ± 4.55 at 28 days). In the crush injury model no significant difference in fibre size was shown between 7 and 28 days and neither a difference between fast and slow type fibres (Fig 2).

### Analysis of the expression of myogenic transcription factors, miRNAs and muscle-specific E3 ubiquitin ligases

**Fig 3:**
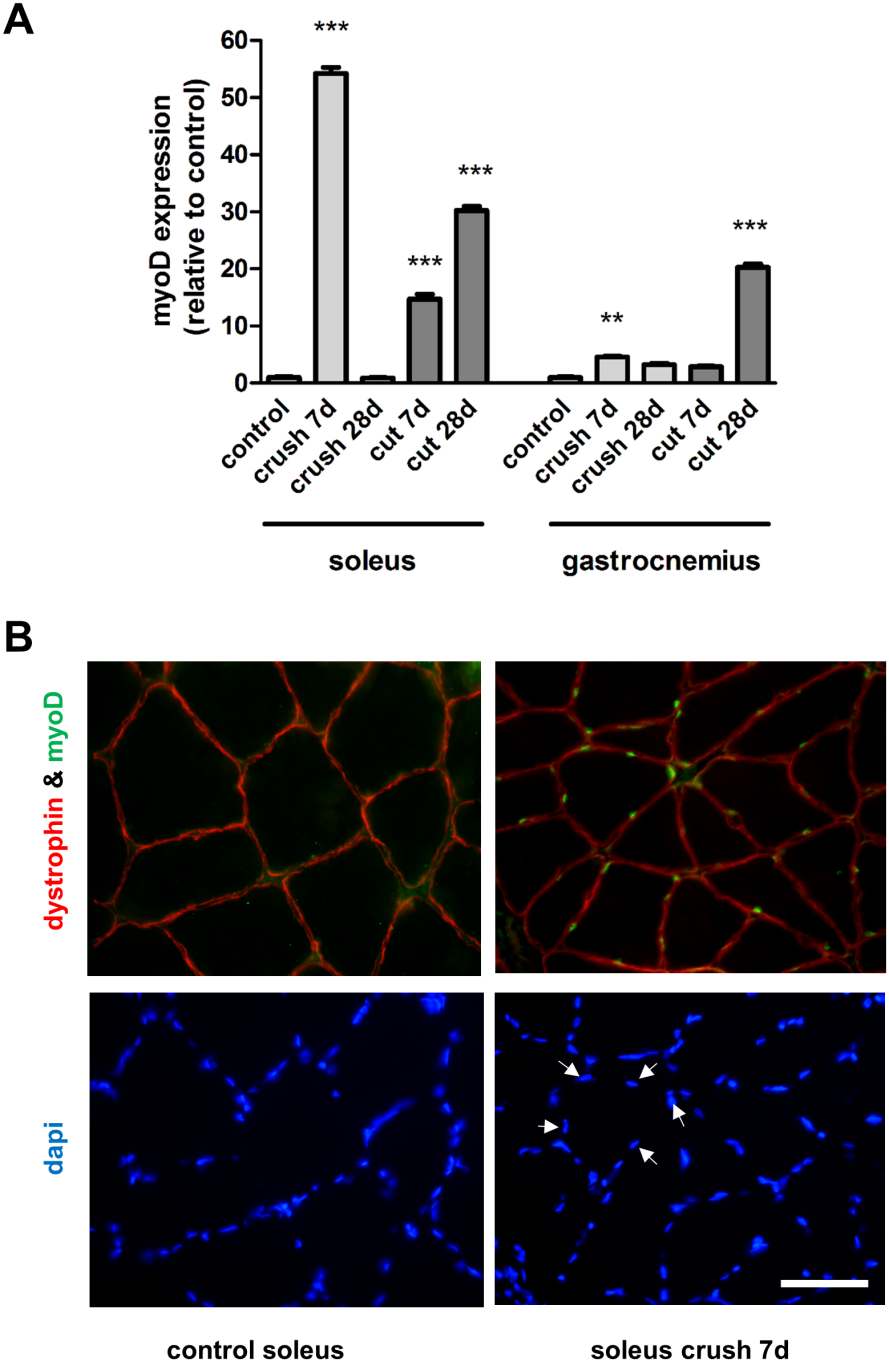
Expression of MyoD in muscles after nerve injury. **(A)** The medial gastrocnemius muscles and the soleus muscles were harvested and fast frozen in liquid nitrogen for subsequent qRT-PCR analysis of myoD expression 7 and 28 days after nerve transection (cut) or crush injury. **P<0.01, ***P< 0.001 represents statistically significant difference to the respective control (unoperated group) muscles. Two-Way ANOVA indicates that the soleus and gastrocnemius muscles are significantly (P<0.001) different for each type of injury. **(B)** Control and soleus muscles harvested from animals 7 days after nerve crush injury were stained with antibodies directed against dystrophin (outline of muscle fibre) and myoD (green). Arrows highlight 5 representative nuclei (DAPI staining) which correspond with myoD positive staining.

**Fig 4:**
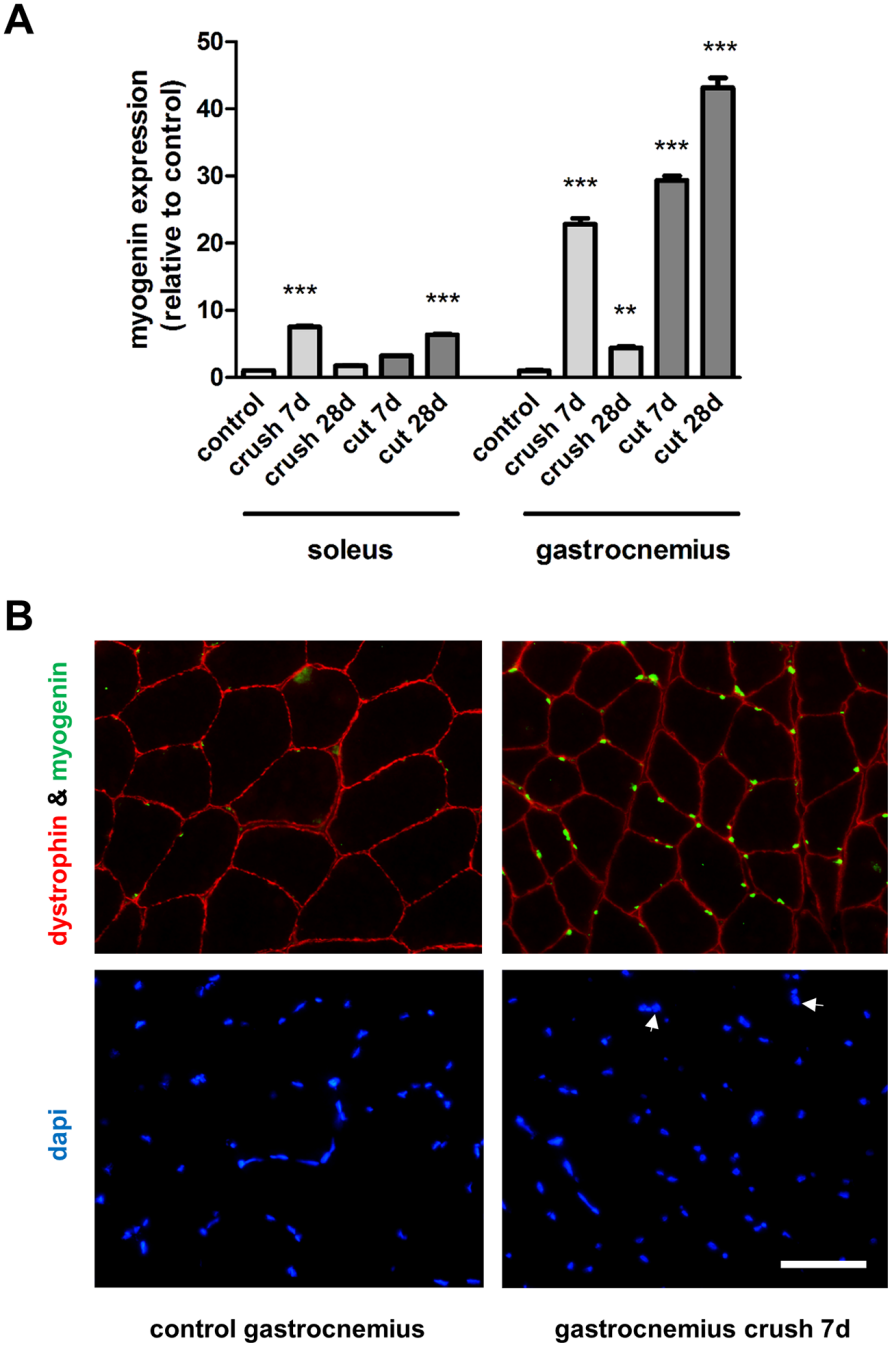
Expression of myogenin in muscles after nerve injury. **(A)** The medial gastrocnemius muscles and the soleus muscles were harvested and fast frozen in liquid nitrogen for subsequent qRT-PCR analysis of myogenin expression 7 and 28 days after nerve transection (cut) or crush injury. **P< 0.01, ***P< 0.001 represent statistically significant differences to the respective control (unoperated group) muscles. Two-Way ANOVA indicates that the soleus and gastrocnemius muscles are significantly (P<0.001) different for each type of injury. **(B)** Control and gastrocnemius muscles harvested from animals 7 days after nerve crush injury were stained with antibodies directed against dystrophin (outline of muscle fibre) and myogenin (green). Arrows highlight 2 representative nuclei (DAPI staining) which correspond with myogenin positive staining.

The expression of the myogenic transcription factors MyoD and myogenin showed a dynamic pattern over time, however with contrasting expression patterns in the different muscle phenotypes. We focussed on two time-points, 7 days (the point after which recovery occurs in crush injured soleus muscles) and 28 days (when maximal atrophy was observed in the transection model). 7 days following crush injury, the expression levels of MyoD were increased 54.23 ± 1.03 and 4.55 ± 0.19 fold in the soleus and the gastrocnemius muscle respectively (Fig 3). 7 days following nerve transection, the expression levels of MyoD were increased 14.74 ± 0.82 and 2.86 ± 0.14 fold in the soleus and the gastrocnemius muscle respectively (Fig 3). At 28 days after injury, the MyoD expression in the soleus muscle returned to near control levels in the crush injury model but was 30.23 ± 0.76 fold higher in the transection injury model (Fig 3). Immunostaining showed that the increased gene expression levels correlated with protein changes—large numbers of MyoD positive nuclei were detected in injured muscles but these were absent in the control muscles (Fig 3). In contrast to MyoD, the reverse gene expression pattern was observed regarding myogenin which was increased 7.51 ± 0.12 and 22.79 ± 0.90 fold in the soleus and the gastrocnemius muscle following crush injury respectively at 7 days, with decreased expression with time (Fig 4). Following nerve transection, there was also a significantly larger increase in myogenin expression in the denervated gastrocnemius versus soleus muscles (Fig 4). Interestingly, myogenin expression was 12.50 ± 0.75 fold higher in the control soleus muscles compared with the control gastrocnemius muscles (S1 Fig). As with MyoD, an elevated number of myogenin positive nuclei correlated with the injury-induced gene expression changes (Fig 4). Taken together these quantitative analyses thus showed a pronounced up-regulation of myogenin in the gastrocnemius muscle 7 days following injury, regardless of injury type, whilst a pronounced up-regulation of MyoD was observed in the soleus muscle 7 days following injury.

**Fig 5:**
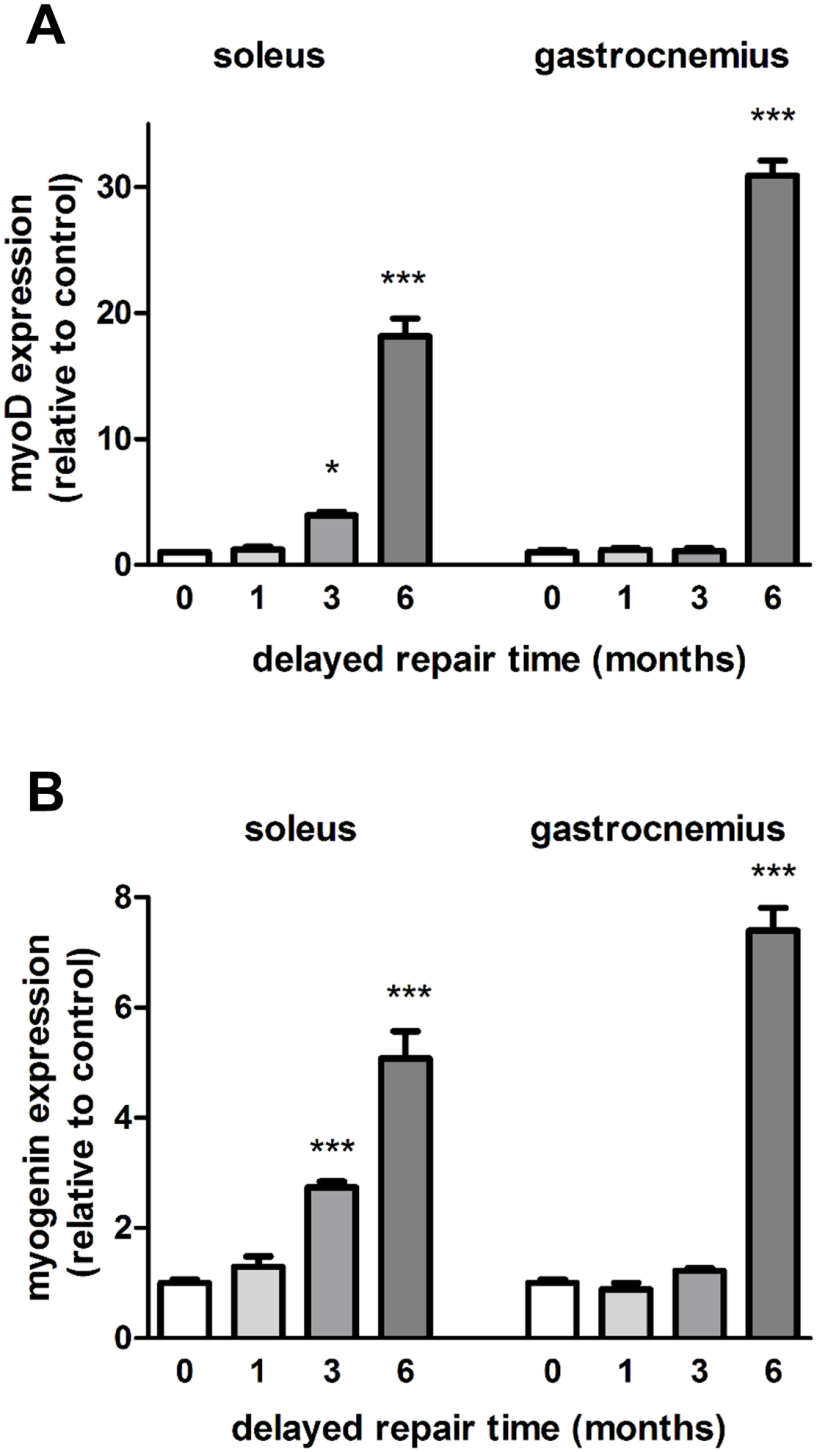
Expression of myogenic transcription factors in the delayed nerve repair model. The medial gastrocnemius muscles and the soleus muscles from ipsilateral operated sides were harvested and fast frozen in liquid nitrogen for subsequent qRT-PCR analysis of **(A)** MyoD and **(B)** myogenin after 0, 1, 3 and 6 month delayed repair with a donor nerve graft. Data are expressed relative to the immediate repairs. *P<0.01, ***P< 0.001 represents statistically significant difference to the respective control (unoperated group) muscles. Two-Way ANOVA indicates that the soleus and gastrocnemius muscles show significantly different expression levels (P<0.01 for MyoD and P<0.05 for myogenin) for each type of injury.

In the delayed nerve repair model, MyoD expression (Fig 5) was up-regulated 3.93 ± 0.28 fold in the soleus muscle after 3 month delayed repair, whilst MyoD was up-regulated only after 6 month in the gastrocnemius muscle, but then to a more extensive degree (30.89 ± 1.19). A similar pattern was observed with myogenin expression which was up-regulated 2.74 ± 0.10 fold in the soleus muscle after 3 months delayed repair, in contrast to the gastrocnemius muscle, which showed only a significant 7.40 ± 0.42 fold increase after 6 months of delayed nerve repair (Fig 5). These quantitative analyses thus showed in the long-term that myogenin and MyoD were significantly up-regulated after 3 month delayed repair in the soleus muscle, in contrast to the gastrocnemius muscle, in which case the myogenic transcription factors was significantly up-regulated only after 6 month delayed nerve repair.

**Fig 6:**
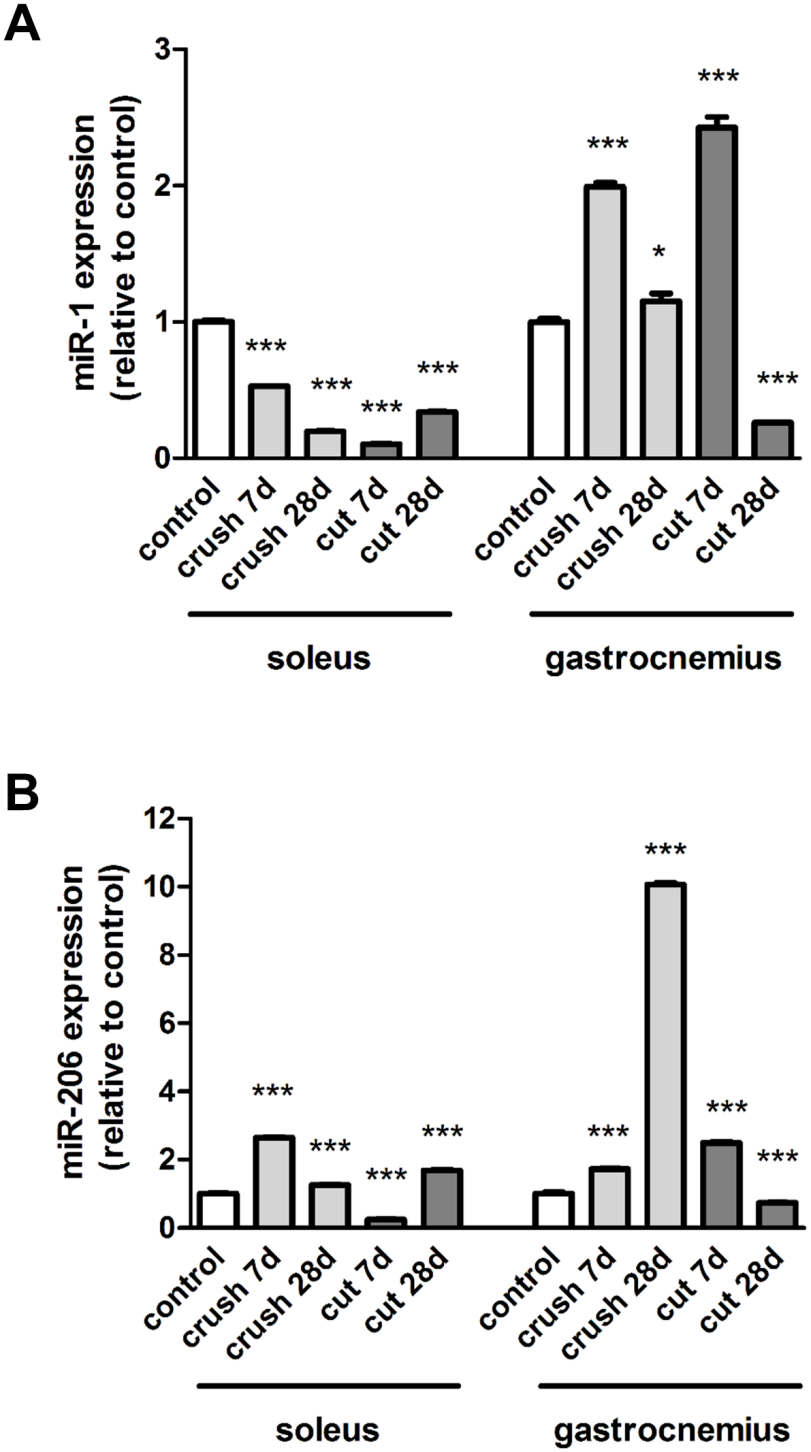
Expression of miRNAs in muscle after nerve injury. The medial gastrocnemius muscles and the soleus muscles were harvested and fast frozen in liquid nitrogen for subsequent qRT-PCR analysis of **(A)** miR-1 and **(B)** miR-206 expression at 7 and 28 days after nerve transection (cut) or crush injury. Data are expressed relative to the control. *P<0.05; ***P< 0.001 represent statistically significant differences to the respective control (unoperated group) muscles. Two-Way ANOVA indicates that the soleus and gastrocnemius muscles are significantly (P<0.001) different for each type of injury.

As with the myogenic transcription factors, miR-1 and miR-206 exhibited opposite expression patterns in the different muscle phenotypes. Following crush injury and nerve transection, miR-1 was increased 1.99 ± 0.03 and 2.43 ± 0.08 fold respectively in the gastrocnemius muscle in comparison to the control at 7 days, whilst the expression was decreased in the soleus muscle (Fig 6). miR-206 was markedly elevated (10.06 ± 0.06 fold) in the gastrocnemius muscle at 28 days following crush injury (Fig 6). Compared to the gastrocnemius muscle, the level of miR-206 in the soleus muscle was significantly 8.20 ± 0.23 fold higher prior to injury (S1 Fig). In contrast, miR-1 expression levels were similar in the control soleus and gastrocnemius muscles (data not shown). In summary, the soleus and the gastrocnemius muscle showed contrasting transcriptional regulation of miR-1 which was down-regulated in the soleus muscle 1 week post injury, regardless of injury type, whilst the gene expression was significantly up-regulated in the gastrocnemius muscle.

**Fig 7:**
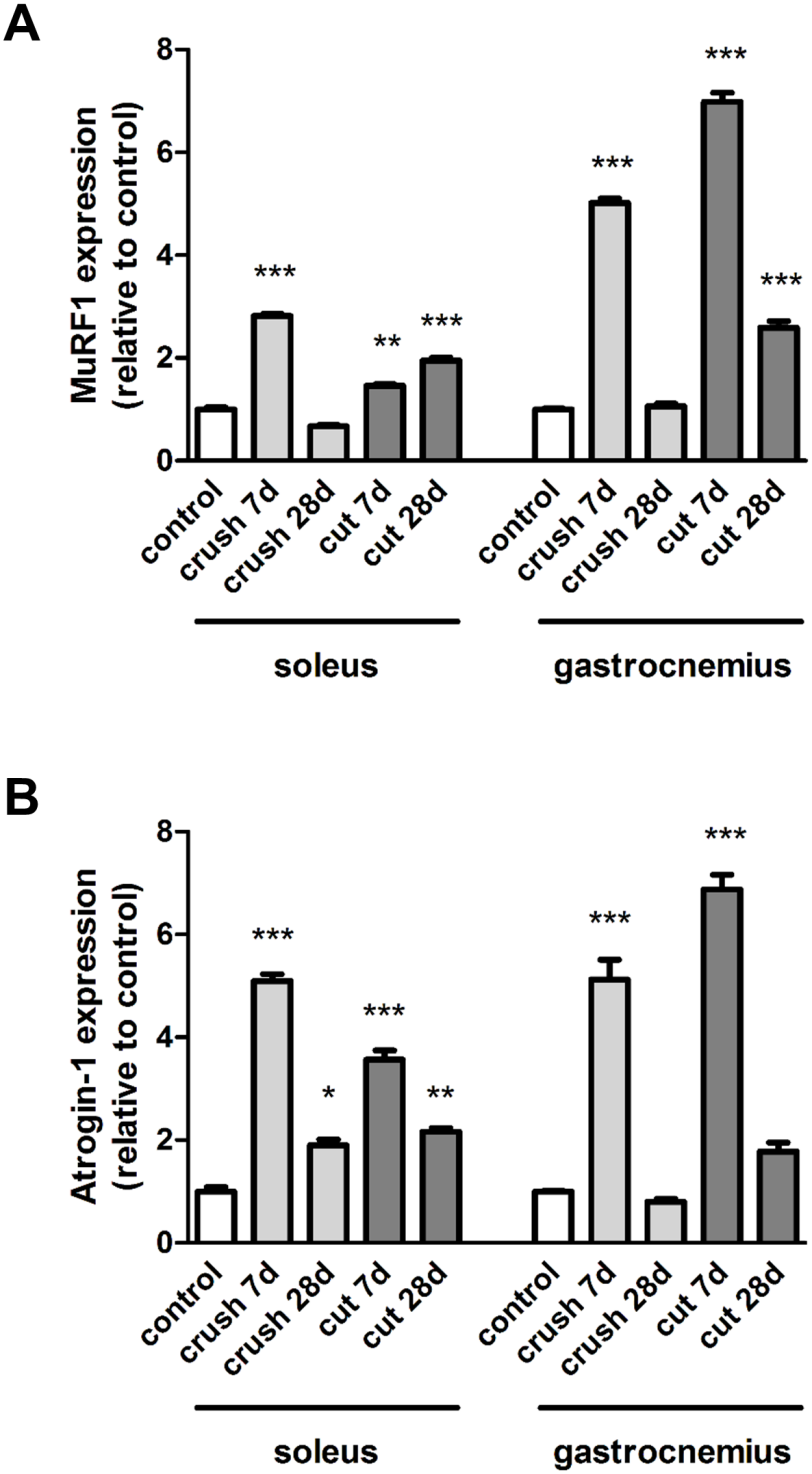
Expression of MuRF1 and Atrogin-1 in muscle after nerve injury. The medial gastrocnemius muscles and the soleus muscles were harvested and fast frozen in liquid nitrogen for subsequent qRT-PCR analysis of **(A)** MuRF1 and **(B)** Atrogin-1 expression at 7 and 28 days after nerve transection (cut) or crush injury. Data are expressed relative to the control. *P<0.05; **P<0.01; ***P< 0.001 represent statistically significant differences to the respective control (unoperated group) muscles. Two-Way ANOVA indicates that the soleus and gastrocnemius muscles show significantly different expression levels (P<0.001 for MURF1 and P<0.01 for Atrogin-1) for each type of injury.

As with the myogenic transcription factors and the microRNAs, the expression pattern of the muscle-specific E3 ubiquitin ligases MuRF1 and Atrogin-1 differed in the two muscle phenotypes. Following crush injury and nerve transection, MuFR1 was increased 5.02 ± 0.09 and 6.98 ± 0.18 fold respectively in the gastrocnemius muscle in comparison to the control at 7 days, whilst only moderate increases were observed in the soleus muscle (Fig 7). Similar data was obtained when Atrogin-1 was analysed following cut injury (Fig 7), levels were increased 6.87 ± 0.29 fold in the gastrocnemius muscle in comparison to the control at 7 days, in contrast to in the soleus muscle, where the increases were lower at 3.56 ± 0.18. Levels of MuRF1 and atrogin-1 were 2.67 ± 0.13 (p<0.001) and 4.03 ± 0.22 (not significantly different) fold higher in the control soleus muscles compared with the control gastrocnemius muscles (S1 Fig).

## Discussion

Despite several studies showing evidence of microRNAs, myogenic transcription and muscle-specific E3 ubiquitin ligases as important players regarding the outcome of peripheral nerve injuries, the exact physiological changes responsible for mediating this effect remains unclear. In this study we have characterised some of these factors with the aim to identify how they are differentially expressed in predominantly fast type (gastrocnemius) and slow type (soleus) muscles which are denervated as the result of two types of nerve injury (crush and transection).

Several studies suggest that muscle phenotype may influence disease progression and a number of clinical studies have reported increased vulnerability of fast fatiguable fibres following peripheral nerve insult. However, the precise molecular mechanisms and signaling pathways that control the expression of the key regulators of muscle protein turnover have not been fully defined. MyoD and myogenin are myogenic transcription factors preferentially expressed in adult fast and slow muscles, respectively [16]. Up-regulation of myogenin in denervated skeletal muscle promotes the expression of acetylcholine receptors [18, 22] and Park et al [7] have demonstrated that myogenin gene transfer into muscle supports spinal cord motors neuron survival and endplate innervation, while myoD gene transfer decrease survival and enhances motor neuron degeneration and muscle denervation.

In contrast with the above mentioned study [7], Moresi et al [19] have demonstrated that myogenin binds and activates the promoter regions of the MuRF1 and Atrogin-1 genes, and adult mice lacking myogenin are resistant to neurogenic atrophy. Histone acetylation has been implicated to affect the denervation-dependent changes in skeletal muscle gene expression [23]; HDAC4 and HDAC5, which repress the expression of Dach2 [22, 23], constitute negative regulators of myogenin [22, 23]. Following nerve injury, several microRNAs are up regulated in an injury dependent pattern, including the muscle specific miRNAs miR-206 and miR-1, which both are transcriptionally regulated by MyoD and myogenin [24]. miR-206 represses the expression of HDAC4 and thus, in accordance to Moresi et al [19], prevents muscle atrophy [19]. In addition, Williams et al [18] demonstrated that miR-206 delays ALS progression and promotes regeneration of neuromuscular synapses in mice, possibly through a HDAC4 dependent mechanism. The mice in which HDAC4 was selectively deleted were reinnervated more rapidly than those of controls following nerve crush or cut. However, Soares et al [25] showed that overexpression of miRNA-206 was sufficient to induce a 10% decrease of fibre size in innervated muscles when compared with controls, which was further confirmed by another experiment showing that inhibition of miRNA-206 was sufficient to induce 10% hypertrophy of innervated muscle.

5´-AMP-activated protein kinase (AMPK), which is a well-known sensor for cellular energy status and metabolic stress, seems to play an important role in the regulation of skeletal muscle mass through the deactivation of the signals in the protein synthesis pathway, mammalian target of rapamycin (mTOR)/p70 S6 kinase (p70S6K) [26] and through the activation of the signals in the protein degradation pathway, Foxo [20] and muscle-specific E3 ubiquitin ligases, such as muscle MuRF1 and MAFbx [21]. Although multiple proteolytic systems are involved in muscle protein breakdown, degradation through the ubiquitin proteasome system (UPS) is indicated to account for up to 80% of the proteolysis during skeletal muscle wasting [27]. The poly ubiquitination is performed by the ubiquitin E3 ligases, which tag ubiquitin to specific protein substrates where MAFbx and MuRF1 are upregulated in multiple models of skeletal muscle wasting [28]. The expression of Atrogin-1 and MuRF1 is, in addition to myogenin [19], regulated by the forkhead box subfamily O (FOXO) transcription factors [29]. Senf et al [29] demonstrated that specific inhibition of FOXO, via expression of a dominant-negative FOXO3a, in rat soleus muscle during disuse prevented 40% of muscle fibre atrophy, demonstrating the importance of FOXO signaling in muscle atrophy. These responses appear to be mediated by a heat shock proteins (HSPs) dependent mechanism [30]. An IGF-1/phosphatidylinositol 3-kinase/Akt axis inhibits FOXO transcription factors through Akt phosphorylation, which result in the retention of FOXO in the cytosol [31], where HSPs bind to and prevent dephosphorylation of AKT and thus prohibit FOXO3a nuclear localization [29]. In addition to MyoD and myogenin [24], miR-1 is transcriptionally regulated by AMPK, where miR-1 appears to mediate an increased protein degradation through HSPs targeting [30, 32].

We demonstrated that the medial gastrocnemius muscle, in comparison to the contralateral side, underwent a much more pronounced atrophy 28 days after injury, compared with the soleus muscle, which correlated with a reduction in muscle fibre size. Furthermore we showed that the soleus and the gastrocnemius muscles exhibited a contrasting transcriptional regulation of miR-1 which was markedly down regulated in the soleus muscle 1 week post injury, whilst the gene expression was substantially up regulated in the gastrocnemius muscle. miR-206 was also markedly elevated in the gastrocnemius 28 days after crush injury. These results are in agreement with previous studies showing evidence of concurrent seed sequences, target genes and expression patterns between miR-1 and miR-206 [24, 33, 34]. In line with our results, Jeng et al [35] showed that the expression level of miR-1 was decreased 1 and 4 weeks following denervation in the soleus muscle. In addition we showed that MuRF1 and Atrogin-1 were up-regulated to a significantly higher extent in the gastrocnemius muscle compared with the soleus muscle.

Adult muscle is highly plastic and can be phenotypically remodelled by intrinsic and extrinsic cues. Sugiura et al [36] demonstrated that functional overload results in transformation of muscle phenotype, from fast fibres to slow-twitch oxidative fibres, in contrast to decreased neuromuscular activity, in which case denervation results in increased expression of all MRFs [37] as well as a slow-to-fast myofibre conversion [38]. In terms of function, MyoD and myogenin differ fundamentally; MyoD functions early in the myogenesis to confer a myogenic fate on mesodermal progenitor cells, whereas myogenin functions later to cause specified myoblasts to differentiate into functional myofibres [39, 40]. Bergstrom et al [41] have demonstrated that MyoD contains specialized domains which are involved in chromatin remodelling. Furthermore, MyoD binds to regulatory regions of genes expressed early in myogenesis and activates their expression [42, 43]. On the other hand, myogenin is thought to bind efficiently within the regulatory regions of genes expressed late in myogenesis after MyoD has performed chromatin remodelling [42–44]. To make it even more complex, the myogenic bHLH regulatory factors need to dimerize with E proteins to form efficient trans-activating heterodimers, after which they can bind to conserved E-box sequences in the regulatory regions of muscle genes [40]. In agreement with earlier studies, the expression of MyoD and myogenin was increased rapidly following injury, there was a pronounced up-regulation of myogenin in the gastrocnemius muscle 7 days following injury, whilst a significant up-regulation of MyoD was observed in the soleus muscle at the same time point. In the delayed nerve repair model, myogenin and MyoD were significantly up regulated after 3 month delayed repair in the soleus muscle, in contrast to the gastrocnemius muscle, which showed significant up regulation of the myogenic transcription factors first after 6 months delayed repair.

So why are slow muscle fibres apparently more resilient to peripheral nerve injury than fast fibres? Earlier research regarding the role of myogenic transcription factors and microRNAs are conflicting. One could hypothesize that the more pronounced atrophy seen in the gastrocnemius muscle following denervation is due to a higher expression level of miR-1 through a muscle-specific E3 ubiquitin ligases dependent mechanism. miR-1 could mediate down-regulation of HSP, followed by dephosphorylation and nuclear translocation of FOXO, and up-regulation of FOXO downstream target genes including MuRF1 and Atrogin-1 with ultimately an increased protein degradation [30, 32]. Although the protein substrates that are ubiquitinated by Atrogin-1/MAFbx and MuRF1 remain largely unknown, some evidence indicates that Atrogin-1/MAFbx ubiquinates and degrades MyoD [45], which in part could explain why MyoD is expressed in a significant lower extent in the gastrocnemius muscle. Another possible explanation for the increased vulnerability of fast fatiguable fibers is that myogenin is expressed in a higher extent in the gastrocnemius muscle compared to the soleus muscle following injury, since myogenin mediates denervation-induced atrophy by binding the promoter regions of the MuRF1 and Atrogin-1 genes [19]. However, as mentioned above, up-regulation of myogenin in denervated muscle promotes the expression of acetylcholine receptors [18, 22, 46] and thus enables a bi-directional signaling between motor neurons and skeletal muscle neuromuscular junctions. Several studies implicate miR-206 in promoting regeneration of neuromuscular synapses and preventing muscle atrophy through a HDAC4 dependent mechanism, raising the possibility that miR-206 represents an inadequate protecting mechanism, and that the soleus muscle is more resilient to injury because the levels of miR-206 are higher prior to injury [18].

One limitation in our study is that there is no direct evidence of a causal link between the myogenic transcription factors, miRNAs and myogenic transcription and muscle-specific E3 ubiquitin ligases. Further investigations, using muscle cells with knockdown or over-expression are necessary for clarification of the underlying mechanisms. Despite considerable surgical innovation, the outcome following peripheral nerve injury is still poor, both in aspect of sensory and motor outcome. Thus, identification of the signaling pathways and cellular mediators of peripheral nerve injury and repair remains a major challenge in the search for novel therapeutics. The muscle specific gene expression is governed not only by the myogenic helix-loop-helix transcription factors. Both phosphorylation, which can modulate the function of the myogenic HLH proteins [47], the presence of appropriate patterns for heterodimer formation [40] and the presence of microRNA are also of importance. Furthermore emerging evidence suggests that microRNA expression can be regulated post-transcriptionally through differential processing of pre-miRNAs [48]. Our study provides further insights regarding the intracellular regulatory molecules that generate and maintain distinct patterns of gene expression in different fibre types following peripheral nerve injury. However, many questions remain unsolved and further work in identifying key muscle biochemical pathways is needed to make progress in the development of muscle directed treatments.

## Supporting Information

S1 Fig Expression of baseline levels of genes in control uninjured gastrocnemius and soleus muscles. Medial gastrocnemius muscles and the soleus muscles were harvested and fast frozen in liquid nitrogen for subsequent qRT-PCR analysis of **(A)** myoD **(B)** myogenin **(C)** miR-1 **(D)** miR-206 **(E)** MuRF1 and **(F)** Atrogin-1. Expression levels in the soleus muscle are compared with the gastrocnemius muscle (normalised to value = 1). ***P< 0.001 represent statistically significant differences to the gastrocnemius muscle.
(TIF)
