# Supplementary figures and images for: Investigation of the Expression of Myogenic Transcription Factors, microRNAs and Muscle-Specific E3 Ubiquitin Ligases in the Medial Gastrocnemius and Soleus Muscles following Peripheral Nerve Injury
Source: PLoS One. 2015 Dec 21;10(12):e0142699. doi: 10.1371/journal.pone.0142699 (PMC4686181; doi:10.1371/journal.pone.0142699)

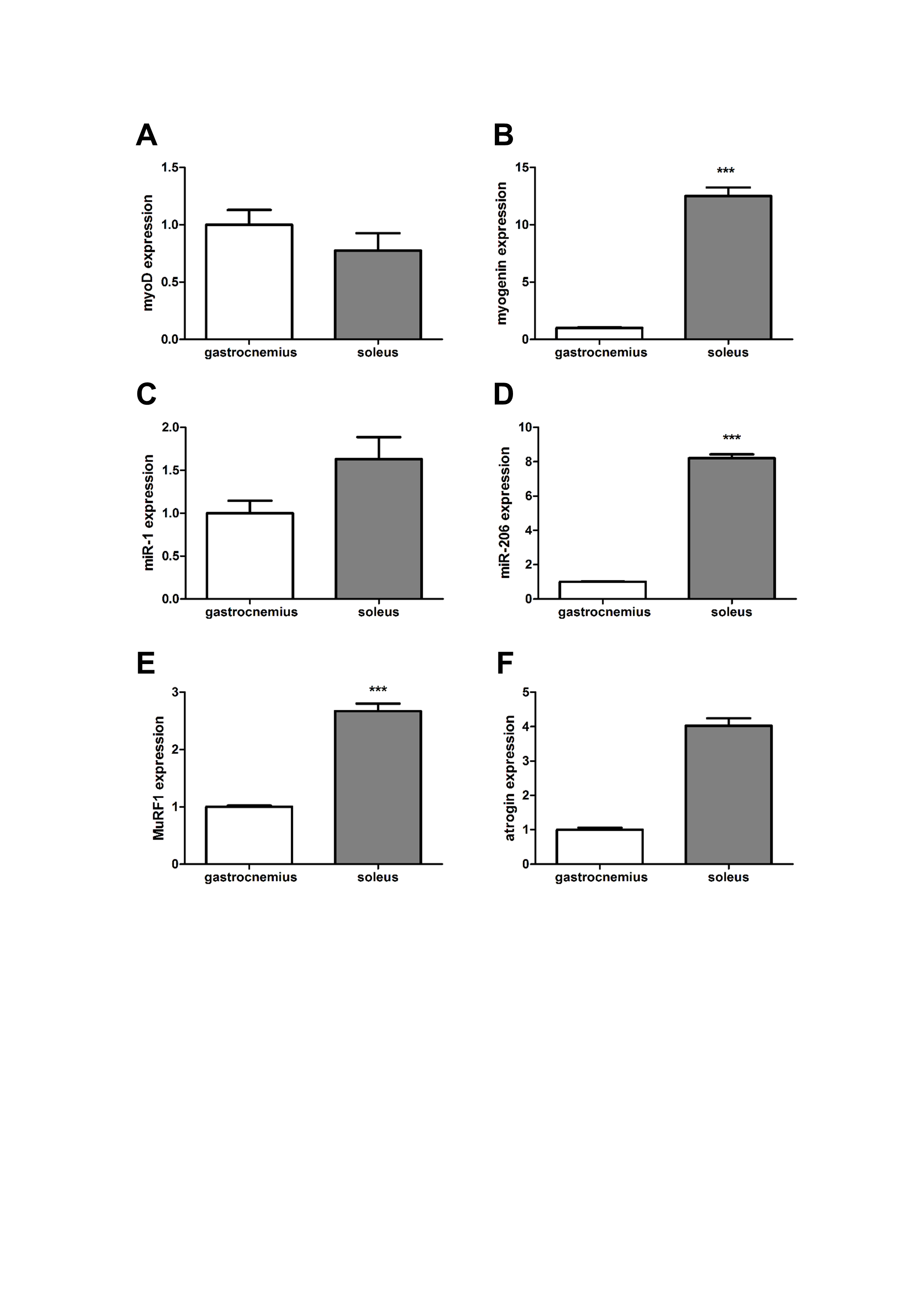

Supplement: S1 Fig — Medial gastrocnemius muscles and the soleus muscles were harvested and fast frozen in liquid nitrogen for subsequent qRT-PCR analysis of (A) myoD (B) myogenin (C) miR-1 (D) miR-206 (E) MuRF1 and (F) Atrogin-1. Expression levels in the soleus muscle are compared with the gastrocnemius muscle (normalised to value = 1). ***P< 0.001 represent statistically significant differences to the gastrocnemius muscle. (TIF) [file pone.0142699.s001.tif]
